# Dissection of a Single Rat Muscle-Tendon Complex Changes Joint Moments Exerted by Neighboring Muscles: Implications for Invasive Surgical Interventions

**DOI:** 10.1371/journal.pone.0073510

**Published:** 2013-08-13

**Authors:** Huub Maas, Guus C. Baan, Peter A. Huijing

**Affiliations:** MOVE Research Institute Amsterdam, Faculty of Human Movement Sciences, VU University, Amsterdam, The Netherlands; West Virginia University School of Medicine, United States of America

## Abstract

The aim of this paper is to investigate mechanical functioning of a single skeletal muscle, active within a group of (previously) synergistic muscles. For this purpose, we assessed wrist angle-active moment characteristics exerted by a group of wrist flexion muscles in the rat for three conditions: (i) after resection of the upper arm skin; (ii) after subsequent distal tenotomy of flexor carpi ulnaris muscle (FCU); and (iii) after subsequent freeing of FCU distal tendon and muscle belly from surrounding tissues (MT dissection). Measurements were performed for a control group and for an experimental group after recovery (5 weeks) from tendon transfer of FCU to extensor carpi radialis (ECR) insertion. To assess if FCU tenotomy and MT dissection affects FCU contributions to wrist moments exclusively or also those of neighboring wrist flexion muscles, these data were compared to wrist angle-moment characteristics of selectively activated FCU. FCU tenotomy and MT dissection decreased wrist moments of the control group at all wrist angles tested, including also angles for which no or minimal wrist moments were measured when activating FCU exclusively. For the tendon transfer group, wrist flexion moment increased after FCU tenotomy, but to a greater extent than can be expected based on wrist extension moments exerted by selectively excited transferred FCU. We conclude that dissection of a single muscle in any surgical treatment does not only affect mechanical characteristics of the target muscle, but also those of other muscles within the same compartment. Our results demonstrate also that even after agonistic-to-antagonistic tendon transfer, mechanical interactions with previously synergistic muscles do remain present.

## Introduction

Transferring the tendon of insertion of a selected muscle to the insertion of another muscle is a surgical intervention performed frequently in a number of clinical conditions (e.g., cerebral palsy, obstetric brachial plexus palsy, stroke, and spinal cord injury), both in the lower extremity [1], and in the upper extremity [2,3]. In cases of paralysis, a tendon transfer aims to restore an important motor function that has been lost by transferring a muscle with intact innervation [4]. In cases of spasticity, a presumed problematic imbalance of muscle forces at a joint, thought to be caused by the hyperactive and uncontrollable muscle overpowering its antagonistic muscles, is corrected [5].

In general, gait or upper extremity function improves after recovery from such surgery and patients report satisfaction with this improvement [6–8]. However, the actual mechanical effects of the (to be) transferred muscle at the joint is seldom evaluated in human patients. Some research groups had the unique opportunity to measure tendon forces of a human muscle just prior to being transferred [9,10]. During tendon transfer surgery, acute effects of disrupting connective tissues on muscular mechanical properties have been reported [5,11,12], indicating effects of epimuscular myofascial force transmission (for reviews, see 13,14). Epimuscular myofascial force transmission is defined as force transmission between a muscle and its immediate surroundings via pathways other than the origin and insertion.

Recently, significant acute effects of tenotomy and partial dissection of flexor carpi ulnaris muscle (FCU) on wrist moment exerted by several wrist flexion muscles were reported for patients with cerebral palsy [15]. However, wrist moment was quantified in neutral wrist position only. When moving the wrist joint, muscle length and muscle relative position are altered. Hence, different mechanical effects of epimuscular myofascial loads are expected [16,17]. Therefore, our first aim was to investigate if effects of FCU tenotomy and muscle-tendon dissection on wrist flexion moment exerted by multiple muscles vary as a function of wrist angle.

Besides severing connective tissue linkages of FCU, tenotomy and dissection involves also disruption of other connective tissue structures (e.g., compartmental fascia) that are linked to adjacent muscles [18,19]. Such fasciotomy yields changes in optimal muscle force and other characteristics of the length-force curve [20,21]. Therefore, we predict that FCU tenotomy and muscle-tendon dissection not only affects the mechanical contribution to joint moment of FCU exclusively, but will alter also moments exerted by neighboring wrist flexion muscles.

To understand functional outcomes of tendon transfer surgeries, an evaluation is needed regarding the contributions to wrist joint moment of transferred muscle being active simultaneously with its previously synergistic muscles. This is particularly relevant, because scar tissue formation, frequently observed following invasive orthopedic surgeries [22,23], is reported to affect the mechanics of transferred as well as of neighboring muscles considerably [24–26]. In addition, several studies in human patients, as well as various animal models (e.g., rats, cats and monkeys) have shown that neural adjustments of motor patterns occur only partially, or do not occur at all [27–31]. As a consequence, activity of transferred muscle remains synchronous with its previously synergistic muscles. Therefore, our second aim was to investigate possible mechanical interaction between transferred FCU and its previous synergistic muscles during coactivation.

As there are severe limitations of studying tendon transfers in humans (e.g., it is not ethical to perform a second surgery for experimental measurements), we recently presented a rat model to study effects of transferring FCU muscle to the distal tendons of extensor carpi radialis brevis and longus muscles (ECR) [24–26]. As discussed in those papers, the model mimics important aspects of the human condition, such as scar tissue formation at the tendon and muscle belly boundaries.

## Methods

### Animals

Male Wistar rats were assigned to either the control group (n=6) or the tendon transfer group (n=6). Surgical and experimental procedures were in strict agreement with the guidelines and regulations concerning animal welfare and experimentation set forth by Dutch law and were approved by the Committee on Ethics of Animal Experimentation at the VU University (Permit Number: FBW 08-01).

### I. Surgical procedures for FCU-to-ECR tendon transfer

Tendon transfer surgery was performed under aseptic conditions, with the rats (body mass at the time of surgery 139±27 g) deeply anesthetized (respiration of 1-3% isoflurane). At the same time, the rats were treated with a single dose (0.03 ml) of buprenorphine (Temgesic®, Schering-Plough, Maarssen, The Netherlands; 0.3 mg/ml solution) for pain relief. Body temperature was monitored and the anesthetic state was checked frequently by evaluating withdrawal reflexes. Surgical procedures have been described in detail previously [25]. In brief, the distal tendon and distal half of the muscle belly of FCU was dissected free and transferred to the tenotomized two distal tendons of extensor carpi radialis (ECR). After recovery from surgery, the rats were kept in their cages for five weeks with access to food and water ad libitum.

### II: Quantification of wrist joint angle-moment characteristics

#### (a): Animal conditions

At the time of assessment of wrist angle-moment characteristics, rat body mass was 334±19 g for the tendon transfer group and 376 ± 37 g for the control group. Prior to surgical preparations for the experiment, the rats were anesthetized deeply, as judged by complete absence of withdrawal reflexes, using intraperitoneally injected urethane (initial dose 1.2 ml/100 g body mass, 12.5% urethane solution). If withdrawal reflexes could be elicited, supplemental doses (0.3-0.5 ml each time) were administered.

The right forelimb was shaved, and the skin was resected from the shoulder to the wrist. Further dissection was performed within the brachium to secure a metal clamp to the humerus for later fixation in the experimental setup.

To prevent hypothermia, the animals were placed on a heated water pad at approximately 37° C. During the experiments, an airflow around the muscle controlled ambient temperature (22 ± 0.5 °C) and humidity (80 ± 2%). Dehydration of forelimb was prevented by regularly irrigating exposed tissues with isotonic saline. At the end of the experiments, without regaining consciousness, the rats were euthanized with a lethal dose of pentobarbital sodium (200 mg i.p.) and double-sided pneumothorax was performed.

#### (b): Experimental conditions

**Figure 1:**
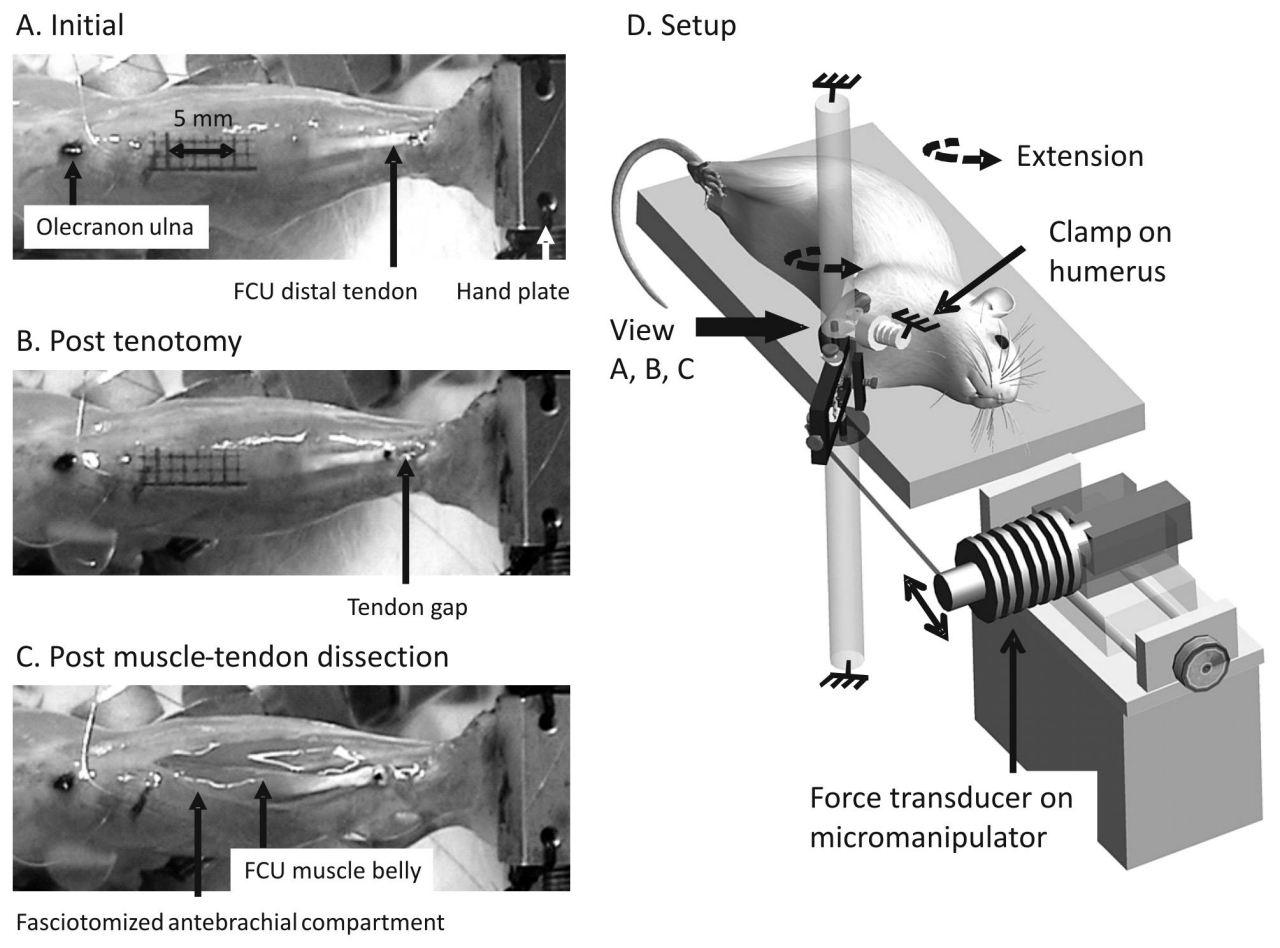
Schematic drawing of experimental setup and FCU imaged in various states of dissection of the right forearm. A-C. Palmar view of a right forearm mounted in the experimental setup (control animal). Measurements were performed in three conditions: (A) After resection of skin of the upper and lower arm (initial condition). Marker on distal tendon indicates intended location of tenotomy, later to be performed. (B) After distal tenotomy of FCU. Note the retracted proximal part of the tendon indicating previous pre-stress in the extended wrist position (22°). (C) After freeing distal tendon and most distal two-third of FCU muscle belly from surrounding tissues, keeping the muscle’s primary blood supply and innervation intact. Obtaining this condition involved nearly full longitudinal antebrachial fasciotomy followed by blunt dissection of connective tissues at the muscle-tendon boundaries. D. Schematic of the experimental setup. The right forelimb was secured by rigidly clamping of the humerus and by tying the hand to a metal plate. The forearm was secured in horizontal position with the elbow joint at approximately 120°. In the experiment, the wrist angle was manipulated by repositioning the force transducer that was attached to a pulley by a Kevlar thread. Each 1-mm translation of the force transducer corresponded to a 5.4° rotation of the hand unit and, hence, a similar change in wrist joint angle. For a detailed description of the apparatus see 26. Scale: Double arrow in (A) indicating 5 mm applies to all panels. Drawing was made in Anim8or version 0.95 (www.anim8or.com).

Within the brachial compartment, the ulnar and median nerves that innervate the wrist and digit flexors [32], were identified and placed jointly in one bipolar cuff electrode. The palmar side of the right hand was tied to a small aluminum plate with 1–0 silk sutures. The right forelimb was secured rigidly to our custom-built apparatus to assess isometric wrist joint moments in the rat (Figure 1D, see also 26). The apparatus consists of a hand fixation unit, which is connected to a force transducer (Hottinger Baldwin, maximal output error <0.1%, compliance of 0.0048mmN^-1^) mounted on a single axis micropositioner. Isometric wrist moments can be assessed at various wrist angles by repositioning the force transducer (1 mm linear translation corresponding to a 5.4° change in wrist angle). With the hand attached, the hand plate was mounted in the hand rotation unit, such that the axis of the apparatus and the wrist’s axis of rotation were aligned. This was verified by minimizing joint moments upon exertion of a load parallel to the forearm to the olecranon of the ulna. Wrist joint moment due to this load was found to be negligible (i.e., ~ 0.1 mNm).

We collected wrist angle-moment data for three successive conditions of FCU (Figure 1A–C):

(i) after resection of the upper arm skin (initial condition, ‘intact’).
(ii) after subsequent distal tenotomy of FCU. Reaching the tendon requires creating a small window in the compartmental fascia, but pilot work (unpublished observations, Huijing and Baan) indicated that this does not affect muscular properties.
(iii) after subsequent freeing of FCU distal tendon and the distal two-third of its muscle belly from surrounding tissues (indicated below as MT dissection). Creating the third condition involved not only blunt dissection of connective tissues at the muscle-tendon boundaries, but also nearly full longitudinal fasciotomy of the antebrachial fascia (see Figure 1C). FCU muscle belly was not fully isolated in order to keep its blood supply and innervation intact and, hence, FCU muscle fibers remained excitable via stimulation of ulnar and median nerves. Previously, we found that in such a state of dissection forces exerted by FCU muscle fibers and, hence their contribution to wrist moment was negligible [26].

The palmar muscles of the antebrachium, including (transferred) FCU, were excited maximally by supramaximal simultaneous stimulation of the ulnar and median nerves via the cuff electrode connected to a constant current source (0.3 mA, pulse width 100 µs). Wrist joint moments were assessed for different wrist joint angles (the neutral position, 0°, being defined as the hand in line with the forearm). More flexed positions of the wrist were defined negative and more extended wrist positions as positive. The wrist angle was changed in steps of 10.8° (i.e., 2 mm steps of the force transducer).

Prior to stimulation of the nerves, the wrist was brought to the target joint angle. Two twitches were evoked, followed by a tetanic contraction of the muscles (pulse train 500 ms, stimulation frequency 100 Hz). After each contraction, the muscles were allowed to recover in neutral wrist position (i.e., zero passive moment) for 2 minutes. Force signals were digitized at 1 kHz and stored on a PC using a data acquisition board (PCI-6221, National Instruments, Austin, TX, USA).

### Comparison with wrist moments of selectively activated FCU

To allow comparison of wrist angle-moments exerted by the wrist flexor muscle group to those of selectively activated FCU (control as well as transferred muscle), we will use part of our data previously reported [26]. These data were obtained in identical individual animals, with one exception: one rat of the tendon transfer group for which FCU moments were not measured, reducing the number of rats (n=5).

Surgical procedures and experimental conditions in that study [26] were identical to those described above, except for those related to selective muscle excitation. A pair of copper electrodes (50 µm diameter) was inserted into FCU muscle belly (approximately 2 mm apart) without additional disruption of connective tissues enveloping the tendons and muscle bellies in the antebrachium. To minimize the likelihood that muscle fibers from adjacent muscles were excited as well, electrodes were inserted near the motor point at which threshold currents are minimal. FCU muscle was excited using currents 2-3 times excitation threshold (constant current 0.3-0.6 mA, pulse width 100 µs). This corresponded to the current at which wrist moment increased only minimally with higher amplitudes, i.e. at which FCU muscle fibers were excited maximally. As much higher currents are required to excite muscle fibers directly rather than through their intramuscular motor nerves, these experimental conditions were considered to yield selective maximal excitation of nerve-endings within FCU muscle only, leaving all other wrist flexors passive.

The wrist was brought to the target joint angle and then two twitches were evoked, followed by a tetanic contraction of FCU (pulse train 500 ms, stimulation frequency 100 Hz). Wrist angle-moment data of FCU were collected for the same states of dissection as described above (see 26). For the present paper, however, the results obtained in the initial condition (Figure 1A) were used exclusively.

### Treatment of data

In this paper, we will focus on active moments, i.e. the added total moment exerted on muscle excitation. This was assessed by subtracting passive moment (the mean for the 50 ms time window just prior to the tetanic contraction) from total moment (the mean for the last 50 ms of the tetanic plateau) at identical wrist angle. In accordance with our definitions of wrist angles, flexion moments were assigned positive values and extension moments were assigned negative values.

In addition, we calculated the difference between wrist moments measured in the initial condition and wrist moments after both FCU tenotomy and MT dissection. This difference will be indicated as the estimated mechanical effect (moment) of FCU muscle.

### Statistics

Two-way ANOVA for repeated measures was used (i) to analyze effects of FCU distal tenotomy and MT dissection on wrist angle-moment characteristics (factors: wrist angle and state of dissection) and (ii) to test for differences between moments of selectively activated FCU and the estimated mechanical effect of FCU (factors: wrist angle and stimulation mode). Bonferroni post hoc tests were performed to test for differences between states of dissection. P values < 0.05 were considered significant.

## Results

### Control group

**Figure 2:**
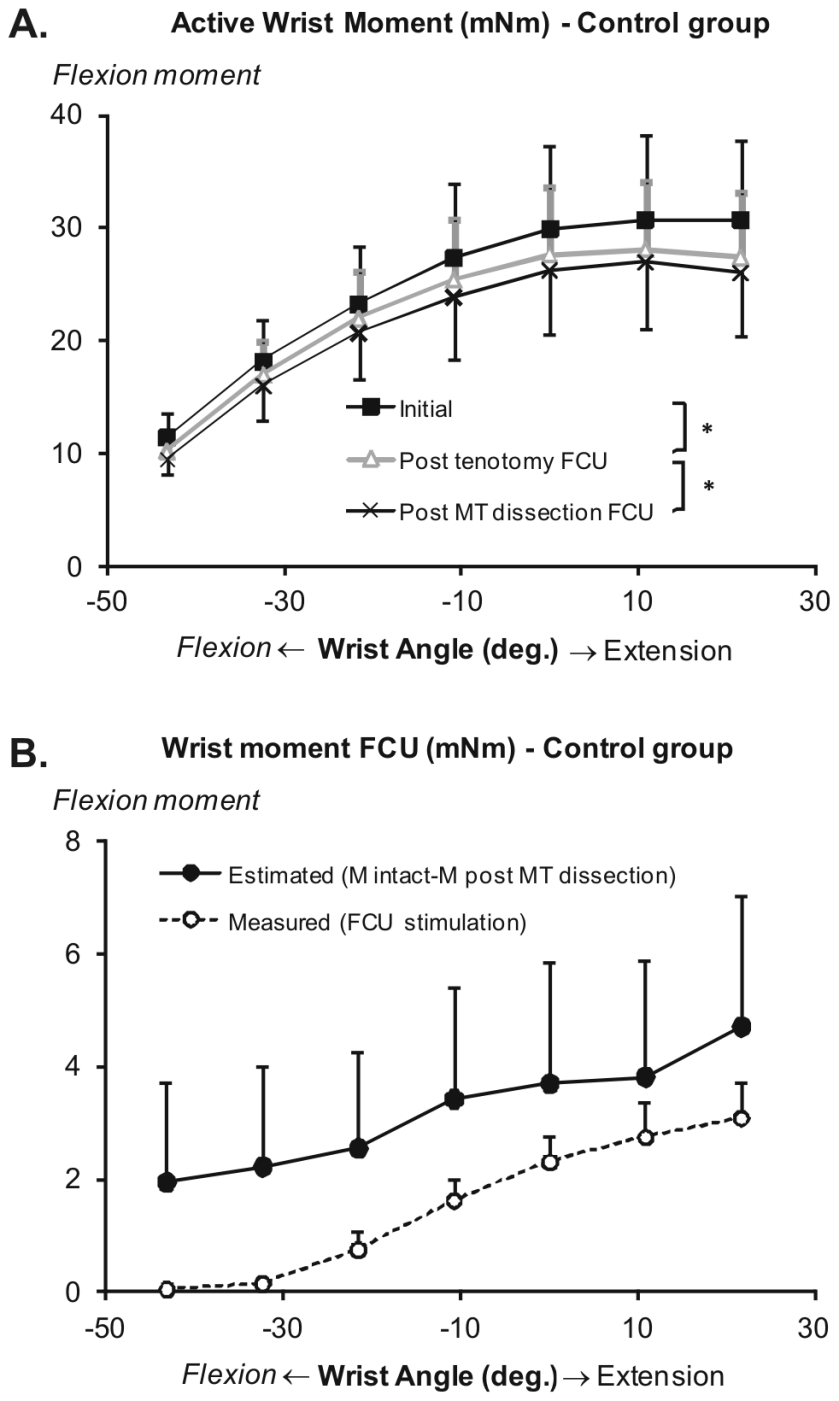
Control group wrist angle-flexion moment characteristics. A. Mean active wrist moment exerted by wrist flexors on simultaneous stimulation of ulnar and median nerves as a function of wrist angle. Asterisk denotes significant differences (p < 0.05) between successive conditions related to state of dissection. Values are shown as mean + or - SD (n = 6). B. Estimated moment of FCU muscle, as calculated by subtracting wrist moments exerted after both FCU tenotomy and MT dissection from moments measured in the initial condition. Dashed line indicates the wrist angle-moment characteristics of selectively activated control FCU muscle in the same group of rats (previously reported [26]). The two curves are significantly different (p < 0.05, ANOVA).

Active moment exerted at the wrist on ulnar-median nerve stimulation varied as a function of wrist angle in a fashion expected from wrist flexors (Figure 2A). Peak flexion moment in the initial condition was 30.8 ± 7.4 mNm and found at 11° wrist extension (Figure 2A). For the range of wrist angles tested, passive wrist moments were low (< 1% of peak active moment, data not shown) and, hence, not considered separately.

ANOVA indicates significant main effects on wrist moment (factors wrist angle and state of dissection). Post hoc analysis indicates (i) significant decreases in wrist moment after FCU tenotomy (mean decrease of 7%) and (ii) a significant additional diminution of moment (by 5%) due to subsequent MT dissection. ANOVA did not indicate significant interaction between factors. This indicates similar changes in wrist moment at each joint angle.

Selectively exciting FCU muscle yields a flexion moment at all wrist angles tested (dashed line in Figure 2B). In the maximally flexed wrist position (at -43°), FCU active moment was near zero (i.e., 0.04 ± 0.01 mNm), while at 11° wrist extension (equal to the optimum wrist angle during ulnar-median nerve stimulation) FCU active moment was 2.7 ± 0.6 mNm.

If the sole effect of FCU tenotomy and MT dissection were elimination of FCU contributions to wrist joint moment, the difference in moment between the ulnar-median nerve stimulation in the intact condition and that following both distal tenotomy and MT dissection should equal the wrist moment of selectively activated FCU. For all wrist angles tested, estimated FCU moments are significantly higher (ANOVA) than the flexion moments of selectively activated FCU (Figure 2B). Note that even in the wrist position at which FCU moment was zero (at -43°), a profound estimated mechanical effect of FCU was found.

The discrepancy between estimated and measured FCU moments indicate that the surgical procedures applied do not only affect FCU muscle, but decrease also the summed mechanical effect of its synergistic wrist flexion muscles. Note that any such additional decrease in wrist moment of one of the wrist flexion muscles other than FCU violates the implicit assumption that such changes can be attributed to FCU muscle contributions.

### FCU tendon transfer group

**Figure 3:**
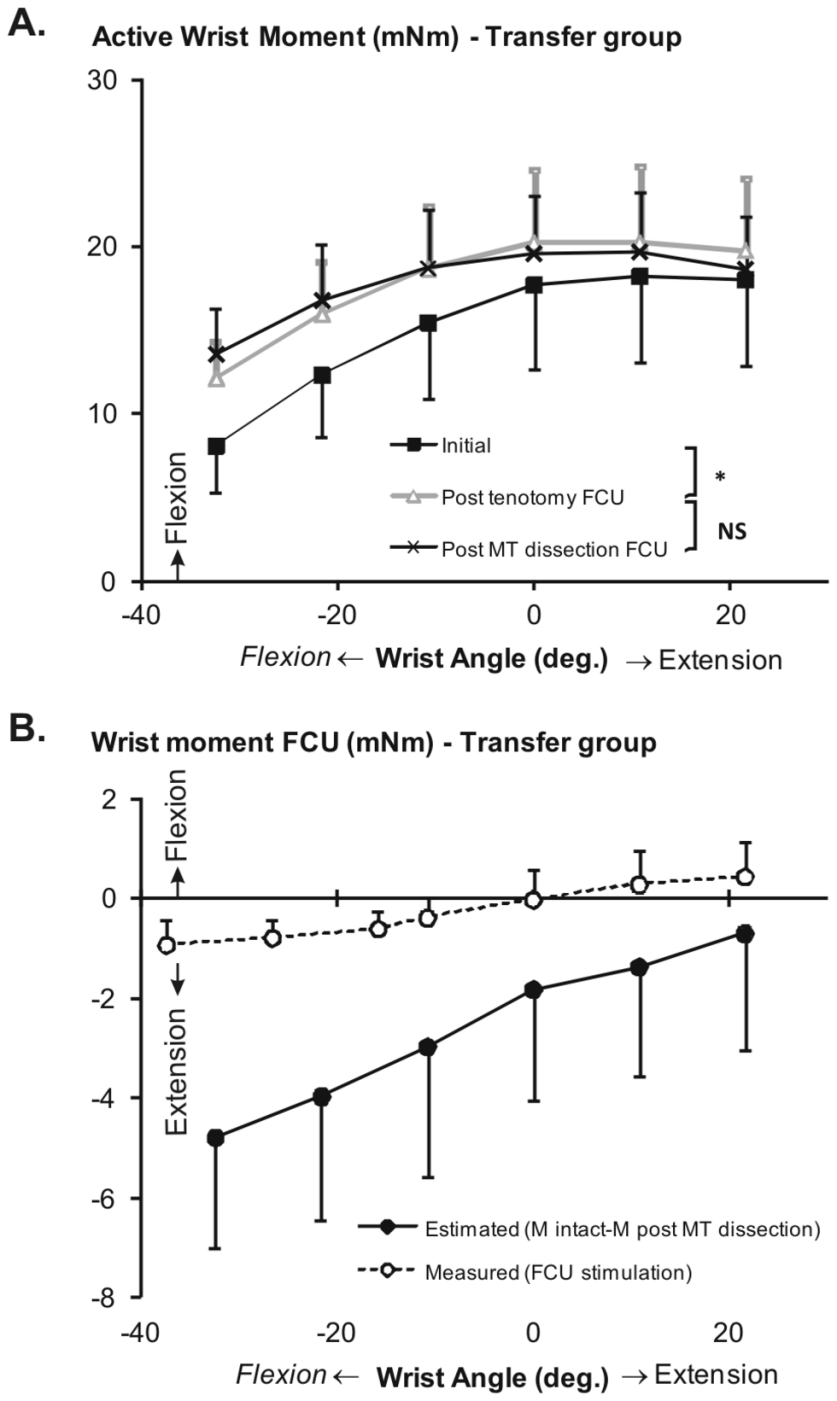
Tendon transfer group wrist angle-moment characteristics. A. Active wrist moment exerted by the remaining wrist flexors and FCU on stimulation of ulnar and median nerves as a function of wrist angle. Asterisk denotes significant differences (p < 0.05) between successive conditions related to state of dissection. NS, not significant. Values are shown as mean + or - SD (n = 6). B. The estimated moment of FCU muscle, as calculated by subtracting wrist moments exerted after both FCU tenotomy and MT dissection from moments measured in the initial condition. Positive values correspond to ﬂexion moments and negative values to extension moments. The dashed line indicates the wrist angle-moment characteristics of selectively activated transferred FCU in five of the same rats (previously reported [26]). The two curves were significantly different (ANOVA).

Post recovery from FCU tendon transfer surgery, active flexion moment was low in the most flexed wrist position and increased with progressive wrist extension up to an optimal value at 11° wrist extension (Figure 3A). Peak flexion moment (18.2 ± 5.2 mNm) was significantly lower than that for the control group (~31 mNm, Figure 2A). Similar to the control group, passive wrist moments were low (< 2% of peak active moment, data not shown).

ANOVA indicates significant main effects on wrist moment (factors wrist angle and state of dissection), as well as significant interaction between these factors. Post hoc analysis located significant increases in wrist moment following tenotomy, particularly at flexed wrist positions (between -32° and -11°, Figure 3A). Peak moment increased to 20.3 ± 4.4 mNm (i.e., equivalent to 111% of the initial condition). Subsequent MT dissection of FCU, however, did not yield any significant additional change of active wrist moment. The fact that post-tenotomy, ulnar-median nerve stimulation-induced wrist flexion moment was enhanced, rather than decreased, indicates that, in accordance with the new path imposed on FCU muscle belly and tendon to its new insertion, transferred FCU exerts an extension moment at the wrist.

Selectively activating FCU yielded bidirectional effects: (a) extension moments in ﬂexed wrist positions and (b) ﬂexion moments in extended wrist positions (Figure 3B). Peak FCU extension moment was 0.9 ± 0.5 mNm and peak FCU flexion moment was 0.6 ± 0.7 mNm.

In contrast, the estimated mechanical effect of transferred FCU, active simultaneously with the unaffected wrist flexors is an extension moment at all wrist angles tested (Figure 3B), being maximal in the most flexed wrist position (5.5 ± 2.6 mNm) and near zero in the most extended position (0.7 ± 2.1 mNm). ANOVA indicates significant main effects (factors wrist angle and stimulation mode), but no significant interaction between these factors. Therefore, also for transferred FCU, estimated moments were profoundly different, both in magnitude and direction, from those obtained if FCU was excited exclusively.

The finding that estimated wrist extension moments of FCU during coactivation of wrist flexion muscles were higher than FCU extension moments exerted during selective excitation suggests that prior to dissection some force was transmitted also from the excited, non-transferred flexor muscles via transferred FCU to the extensor side of the wrist.

## Discussion

This paper addresses two questions aimed at providing insight into the mechanical functioning of a single skeletal muscle active within a group of (previously) synergistic muscles. We found similar effects of FCU tenotomy and MT dissection on wrist moments of the control group at all wrist angles tested (aim 1). Following FCU-to-ECR tendon transfer, wrist angle–moment characteristics were affected by tenotomy, but not by MT dissection. Wrist moments increased significantly, but at flexed wrist positions exclusively (aim 2). The discrepancies between estimated and measured FCU moments clearly prove that the surgical procedures required to perform a tenotomy and MT dissection do not only affect force transmission from control or transferred FCU.

As a limitation of our experimental design could be considered the fact that the distal two-third and not the whole FCU muscle belly was dissected free from its surroundings, since further FCU dissection may also cause enhanced effects on neighboring muscles (see below). Therefore, our approach could have lead to an underestimation of the estimated FCU moment. However, full isolation of FCU was not possible, because this would involve denervating this muscle, a condition not compatible with our goals. In our previous study, we have found that wrist moment of selectively excited FCU with only its proximal one-third still connected to adjacent structures was negligible [26]. Therefore, it is argued that not fully isolating FCU did not alter the major results of the present paper.

### Epimuscular myofascial effects on control FCU

Present effects of tenotomy and MT dissection in the control group are in agreement with those reported for patients with cerebral palsy tested exclusively for the neutral position of the wrist (corresponding to 0° in Figure 2) [15]. Our results extend those observations by showing decrements in wrist moment as a result of post-tenotomy MT dissection, also for more flexed and extended wrist positions. Such a result was interpreted by de Bruin et al. [15] as proof of a contribution from muscle fibers within FCU, still located among the wrist flexors of the antebrachium, to wrist flexion moment after distal tenotomy. Force is then transmitted from FCU via epimuscular myofascial pathways and synergistic muscles to the skeleton. Although this is a plausible explanation, two aspects of our results indicate that additional explanations should be considered as well: (i) The combined effects of FCU tenotomy and MT dissection on wrist moment were significantly higher than the maximal moment exerted by selectively active FCU muscle (Figure 2B); (ii) Effects of MT dissection were found also at the most flexed wrist angles for which moments exerted by selectively activated FCU were found to be near zero. These results indicate that the effects of MT dissection were not limited to disrupting force transmitting pathways of FCU muscles fibers.

Previous studies have shown that muscle force decreases by 10% following partial fasciotomy of dog anterior crural fascia [20] and by up to 14% following full fasciotomy of rat anterior crural fascia [21]. Fasciotomy of the distal two-third of rat antebrachial compartmental fascia, simulating the surgical procedures for FCU tendon transfer in humans, decreased FCU force by up to 40% [33]. Note that these reported changes in muscle force were the result of fasciotomy exclusively, thus, without further MT dissection. In the present study, nerve stimulation-induced active wrist moment decreased by 11-17% as a result of tenotomy and MT dissection. MT dissection in the present study involved also fasciotomy of the antebrachial fascia. Therefore, we conclude that combined surgical procedures, applied to eliminate the contribution of FCU, do affect also active forces exerted by the other wrist flexion muscles.

For direct assessment of the effects of fasciotomy, this intervention should be studied in isolation. In the present study, a tenotomy was performed first. Tenotomy does not only eliminate the myotendinous pathways of muscular force transmission, but causes also enhanced muscle belly shortening during contraction (Figure 1B, see also 11,34). Such lower length of FCU muscle fibers and, hence, decreased forces exerted by them (if on the ascending limb of their length-force curve) will by itself yield decreased wrist flexion moments. This was assessed in our previous study to be a decrease of 93% at the optimum angle [26]. Therefore, the experimental approach used here cannot be applied to validly quantify the contribution of epimuscular myofascial force transmission for a single muscle.

### Effects of mechanical connectivity on transferred FCU

In the present study, we evaluated the mechanical effects of FCU muscle, with its insertion transferred to the extensor side of the hand, when excited simultaneously with its previously synergistic wrist flexors. An adequate neural response to an agonist-to-antagonist tendon transfer requires that timing of activity of the transferred muscle is adapted to its altered mechanical function. Several studies, however, have shown that such neural adaptation occurs only partially, or does not occur at all [27–31]. Consequently, coactivation of transferred muscle and its previously synergistic muscles during limb movements is a relevant condition to study.

Our results indicate that during such coactivation, part of the forces exerted at the dorsal side of the hand and, thus producing an extension moment at the wrist, originates from the unaffected wrist flexion muscles (Figure 3). It should be noted that not the whole FCU is transferred to the extensor side. The origin and the most proximal muscle belly part of FCU remains surrounded by and connected to wrist flexion muscles. In previous studies, we have shown that force transmission between transferred FCU and non-transferred, antagonistic palmaris longus muscle is feasible [24,26]. Indirect evidence for a similar phenomenon has been reported by Riewald and Delp [35], who found that stimulation of rectus femoris muscle following transfer to a flexor site of the knee yielded a knee extension moment. This is most likely the result of force transmission from rectus femoris muscle fibers to the non-transferred neighboring knee extensors. There is ample evidence that such mechanical interactions between adjacent muscle bellies are mediated by connective tissue linkages [13,14,36].

Force transmission from wrist flexors to the extensor side is most pronounced (i.e., the difference between measured and estimated FCU wrist moment is highest) if the wrist is flexed (Figure 3). This can be explained by the lengths of flexion and extension muscles, as well as by the orientation of connective tissue linkages between transferred FCU and its surroundings. If the wrist is flexed, transferred FCU and all other wrist extensors are at a high length, providing a stiff pathway for force transmission. Connections between flexors via FCU to extensors will be lengthened and directed towards the extensor side of the wrist. Thus with the wrist flexed, forces exerted by wrist flexion muscles are more likely to be transmitted to the extensor side (for a detailed description, see 26). The opposite is the case if the wrist is extended. Transferred FCU is linked mechanically to structures at both the dorsal and ventral side of the forearm via physiological and scar tissues [25]. This provides a stiffer than normal mechanical linkage between wrist flexion and extension muscles.

### Concluding remarks

To the best of our knowledge, this is the first study investigating mechanical effects of transferred muscle during coactivation of its formerly synergistic muscles. Taking the important differences between rats and humans (see 25) into account, our results have two clinical implications: (i) Dissection of a single muscle in any surgical treatment does not only affect the target muscle, but also other muscles within the same compartment (ii). Even after agonistic-to-antagonistic tendon transfer, mechanical interactions with previously synergistic muscles can remain. These insights may help understanding the functional outcomes of tendon transfer surgeries, as well as other surgical interventions involving muscle-tendon dissection, which is requisite for improvement of current treatment strategies.
